# Measuring intercultural competence—A review and introduction of the Cult Euro 1 test for intercultural competence

**DOI:** 10.3389/fpsyg.2025.1621499

**Published:** 2025-09-23

**Authors:** Petia Genkova, Henrik Schreiber

**Affiliations:** ^1^Faculty of Business and Social Sciences, Osnabrück – University of Applied Sciences, Osnabrück, Germany; ^2^Department for Psychology and Psychotherapie, University Witten/Herdecke, Witten, Germany

**Keywords:** intercultural competence, psychological assessment, cultural intelligence, measurement validity, situational judgment test

## Abstract

Intercultural Competence is a key factor in ensuring successful interaction between individuals from different cultural backgrounds, particularly in increasingly diverse and globalized societies. Despite its relevance, there is still no consensus on its definition, modeling, or measurement, especially from a psychological perspective. This paper provides a comprehensive review of the theoretical foundations of intercultural competence, including anthropological and psychological definitions of culture, interculturality, and competence. Central psychological models, such as the identity, learning theory, and stress approaches, are introduced as frameworks for understanding the development of intercultural competence. In addition, the paper analyses the conceptual distinctions between competence, personality, and intelligence and discusses their implications for the empirical assessment of intercultural competence. Various existing models and measurement instruments are evaluated in terms of validity, reliability, and cross-cultural applicability. Building on these insights, the paper presents Cult-Euro-1, the first standardized, psychometrically validated test designed to assess both general and German culture-specific intercultural competence. This modular, multi-methodological instrument addresses key conceptual and methodological gaps in the field. The test's practical applications in human resources, education, and social integration are discussed, with an emphasis on its potential to foster intercultural understanding, reduce discrimination, and enhance the participation of migrants in German society.

## 1 Introduction

Due to the advancing global interdependence of the economy and the increasing social diversification, the ability to act efficiently and appropriately in intercultural situations is an important factor for professional success and well-being of the people involved (Deardorff and Arasaratnam-Smith, 2017). The aim of fields of research such as intercultural psychology and intercultural communication is therefore to facilitate and promote “successful” interactions between people from different cultures. Such interactions are expected to prevent social conflicts, facilitate understanding in situations of cultural overlap, and contribute to the management of social integration processes. However, intercultural interactions are often characterized by misunderstandings, prejudices or discrimination and perceived as stressful (Thomas, 1993; Thomas and Utler, 2013).

To overcome these problems, research is being conducted on intercultural competence – the ability to deal with cultural differences, to empathize with people from other cultures, and to interact with them effectively and appropriately (Genkova, 2019). Numerous studies on intercultural competence are based on a wide variety of definitions and predictors (Spitzberg and Changnon, 2009). This is plausible because intercultural psychology/communication is an interdisciplinary meta-discipline composed of different human and social sciences. Research therefore focuses more on the interpretation and discussion of findings and less on diagnostics *per se*. However, it is difficult for researchers and practitioners to find the most suitable assessment method due to the large number of theories, models and measurement instruments.

This article aims to provide an overview of the current state of research on the measurement of intercultural competence, and to present implications and recommendations for action. For this purpose, the concept of competence and the understanding of interculturality in psychology are first introduced and differentiated from related constructs. Building on these considerations, the following section deals with definitions and models, in particular different approaches used in modeling intercultural competence. Subsequently, the measurement of intercultural competence is examined in more detail, and various measurement methods used for the assessment of intercultural competence are discussed. Based on the theoretical explanations, we discuss established and more recent approaches to measure intercultural competence and derive implications for future research. Finally, the Cult-Euro-1 test is introduced, which overcomes the methodological and conceptual weaknesses of previous measurement instruments and can therefore be regarded as particularly valid.

## 2 Classification and differentiation of intercultural competence

Intercultural competence enables effective and appropriate interactions with people from different cultural backgrounds (Thomas and Simon, 2007). Despite its relevance, there is still no consensus on how it should be defined, modeled, or measured (Genkova, 2019; Lonner et al., 2020). The terminology itself varies widely—terms like multiculturalism, cultural adaptation, intercultural sensitivity, and cultural intelligence are often used interchangeably without clarification (Fantini, 2009; Wolff, 2017). This conceptual ambiguity stems from the field's interdisciplinary roots.

Early research, largely shaped by humanities disciplines such as anthropology, ethnology, and philosophy, emphasized cultural understanding in the context of foreign language education and conflict reduction, especially during the Cold War (Genkova, 2019; Helmold, 2020). These approaches focused on cultural knowledge rather than empirical, behavior-based analysis. Over time, it became evident that these humanistic perspectives intersect with psychological theories—such as those concerning stereotypes, prejudice, and communication. Today, psychology is the dominant discipline in intercultural competence research. Accordingly, we examine the constructs of culture, interculturality, and competence from a psychological standpoint, considering the respective roots in other disciplines.

### 2.1 Culture and interculturality

Theories of culture originated in anthropology. Tyler (1871; see Kroeber and Kluckhohn, 1990) defined culture as a complex of knowledge, beliefs, art, laws, customs, and habits shared within a society. A widely cited definition from Geertz (1973) describes culture as a historically transmitted pattern of meanings embodied in symbols, which provides a framework for interpreting the world and guiding action. From this perspective, culture is not innate but learned, socially transmitted, and deeply embedded in collective rituals, language, and worldviews. Anthropological approaches generally resist reductionist interpretations, instead emphasizing the contextual, dynamic, and interpretive nature of culture as a system of shared understandings.

These anthropological perspectives continue to inform psychology but differ in focus. While anthropology emphasizes cultural traits, psychology centers on individuals' behavior and experiences (Genkova, 2019). Culture is viewed as a shared system of values, beliefs, norms, and practices that shapes how individuals perceive, evaluate, and respond to their environment. Triandis (1994), a central figure in cross-cultural psychology, described culture as a set of shared meaning systems transmitted across generations, which influence social behavior and psychological functioning. Berry (1990) emphasizes the role of culture in shaping psychological adaptation and acculturation processes, highlighting the interplay between individual functioning and collective norms. Thomas (1993) defined culture as a system of orientation that informs perception, judgment, and behavior. His concept laid the foundation for many later models of intercultural competence by focusing on culturally shaped action tendencies. These psychological approaches not only enable the empirical study of culture but also underpin models for understanding and assessing intercultural adaptation, effectiveness, and competence. Culture thus serves as a framework enabling individuals to adapt to their environment.

A key challenge in intercultural and cross-cultural psychology is the difficulty of understanding people with fundamentally different cultural orientation systems. As Thomas (1993) notes, we often perceive others through our own cultural lens. Three paradigms address this issue: (1) Absolutism assumes only quantitative differences in cognition across cultures. (2) Relativism argues cultural context so deeply shapes perception that cross-cultural understanding is nearly impossible (Berry et al., 2002). (3) Universalism posits that basic psychological functions are shared across cultures, though expressed differently (Fontaine, 2011). This last view supports efforts to examine measurement equivalence and assess the generalizability of findings (Genkova, 2019).

Intercultural encounters can be stressful, as norms around perception and behavior often clash. Intercultural psychology distinguishes three key approaches to understanding such overlap: the identity approach (acculturation), the learning theory approach (cultural learning), and the stress approach (cultural adaptation; Genkova, 2019).

The identity approach describes how individuals and groups adjust to each other through contact with other cultures. At the group level, acculturation alters collective cultural norms (Berry and Sam, 1997); psychologically, it involves individual-level changes in behavior, identity, and emotional response. Migrants, for instance, vary in how much they adopt host culture practices (Ward and Szabó, 2019). These changes can be both psychological and sociocultural (Searle and Ward, 1990; Jaeger, 2009).

The learning theory approach emphasizes acquiring skills needed for effective intercultural interaction. It assumes that familiar behaviors may fail in a foreign cultural context, even among previously well-adapted individuals. Migrants often need to relearn appropriate behaviors, as existing social norms no longer apply (Genkova and Riecken, 2020). Cultural adaptation, then, is a learning process that varies in speed and effectiveness between individuals.

The stress approach examines how people respond to the psychological challenges of intercultural experiences. Stress is defined as a reaction to internal or external change (Schmidt, 2021), with migration frequently cited as a major stressor. Intercultural encounters can provoke such profound adjustment difficulties that they lead to early termination of international stays or even mental health issues, such as depression. In the 1980s, migrants were overrepresented in psychiatric clinics (Genkova and Riecken, 2020), and extreme forms of intercultural stress are termed “culture shock” (Oberg, 1960).

Successful intercultural behavior can thus be seen in individuals' mental and physical wellbeing, sociocultural adjustment, and professional or academic success abroad (Thomas and Simon, 2007; Ng et al., 2017). Given the importance of intercultural competence in contexts like business, migration, or international cooperation, it becomes essential to identify the individual characteristics that promote success in such settings.

Among these characteristics, competence and personality are frequently studied, with competence proving more predictive of successful intercultural behavior, though general cognitive ability remains the strongest predictor overall (Schuler and Prochaska, 2000; Kanning, 2019). Thus, focusing on competence offers a promising path for understanding and promoting effective intercultural interaction. But what exactly constitutes competence?

### 2.2 Competence

The concept of competence is especially relevant when performance is assessed from a practice-oriented perspective (Hartig and Klieme, 2006). It plays a central role in educational research and the development of educational standards (Klieme et al., 2000). Weinert (2001) defined competence as a general cognitive and context-specific performance disposition, encompassing knowledge, skills, or routines, that enables individuals to manage a range of tasks and challenges. This also requires motivation to engage with complex demands and supports the acquisition of further competences.

Competence differs fundamentally from personality traits, which are relatively stable across time and situations (McCrae and Costa, 2004). While personality may shift due to critical life events (Tracy-Ventura et al., 2016), it cannot be deliberately taught or trained. Traits like extraversion may support the development of certain competences, such as navigating intercultural interactions, but are not competences themselves. Competencies, by contrast, are reflected in observable behavior and are assessed in terms of specific life situations (Erpenbeck, 2012; Genkova, 2019).

Unlike intelligence, which is per definition independent of prior learning, competences are domain-specific, functional, and goal-oriented (Weinert, 2001). Intelligence reflects a general cognitive capacity that is traditionally seen as genetically predisposed. While some controlled studies report modest IQ gains under intensive training, larger meta-analyses find little evidence of generalization beyond trained tasks (Gobet and Sala, 2022; Protzko, 2017). Variations in intercultural competence, for example, may stem from differences in pattern recognition, behavioral adaptation, or language ability, factors linked to context, rather than innate cognitive processing.

#### 2.2.1 Excursus: measuring intelligence

Measuring intelligence differs fundamentally from measuring competence. While the terms *intelligence* and *cognitive performance* are often used interchangeably, a key distinction lies between *fluid intelligence*, the speed, accuracy, and coordination of cognitive processes, and *crystallized intelligence*, or acquired knowledge (Cattell, 1963; Horn and Noll, 1997). Fluid intelligence is strongly linked to academic performance (Deary et al., 2005), career success (Kanning, 2019), learning speed (Gottfredson and Deary, 2004), and even health outcomes (Batty et al., 2007).

In Germany, the Intelligence Structure Test (IST 2000 R; Liepmann, 2007) is a widely used instrument. It assesses verbal, numerical, and figural-spatial intelligence across eleven task types, and includes memory tasks. An optional 40-m module measures crystallized intelligence. Compared to matrix tests, the IST-2000-R captures a broader range of cognitive abilities. Validation studies confirm its strong construct and criterion validity (Kersting and Palmer, 2017).

However, intelligence measurement is inherently culture-bound. Although intended to reflect general cognitive ability, tests are influenced by cultural variables. While verbal and numerical tasks clearly depend on language and prior knowledge, figural-spatial items, such as matrix tests, were long assumed to be culture-fair. Yet, cognitive styles, visual processing, symbolic interpretation, and test familiarity vary across cultures (Genkova, 2019; Gonthier, 2022).

Thus, cross-cultural intelligence assessments must consider cultural context to ensure fairness, just as in measuring intercultural competence. As Gonthier (2023) notes, test design elements like media, symbols, and response formats affect results. Selecting a culturally fair intercultural competence test requires a model that fully incorporates these cultural nuances.

## 3 Definition and modeling of intercultural competence

Drawing on psychological concepts of culture, interculturality, and competence, intercultural competence is defined as a “necessary prerequisite for appropriate, successful and entirely satisfactory communication, encounters and cooperation between people from different cultures” (Thomas, 2002, p. 174). It emerges through learning and development and requires a willingness to engage respectfully with unfamiliar cultures.

An interculturally competent individual possesses nuanced knowledge of both their own and others' cultural characteristics and can respond appropriately by comparing cultural frameworks. This includes the ability to flexibly navigate differing orientation systems and apply learned strategies to new cultural contexts, demonstrating creativity, adaptability, confidence, and emotional stability (Thomas, 2002; Thomas and Utler, 2013).

Over thirty existing models of intercultural competence describe more than 300 sub-competences (Leung et al., 2014). These models vary in dimensional structure (Schnabel et al., 2014, 2015; Wolff, 2017), but common dimensions include tolerance of ambiguity, empathy, perspective-taking, flexibility, communication skills, cultural awareness, openness, and respect (Schnabel et al., 2014). Rather than representing a distinct type of competence, intercultural competence is often viewed as a contextual application of general competence in culturally diverse settings (Bolten, 2007; Wolff, 2017). A review of modeling approaches is therefore essential for its valid measurement.

### 3.1 Modeling of intercultural competence

Taxonomies of intercultural competence models distinguish between trait-oriented and development-oriented approaches (Spitzberg and Changnon, 2009; Bartel-Radic and Giannelloni, 2017). Trait-oriented models include list models (e.g., Bolten, 2007), structural models (e.g., van Dyne et al., 2009), and causal path models (e.g., Deardorff, 2009), which define and relate specific characteristics to distinguish competent from incompetent individuals. In contrast, development-oriented models describe stages of growth (e.g., Bennett, 1986) or cultural adaptation over time (e.g., Ward et al., 2001).

#### 3.1.1 List and structural models

List models conceptualize intercultural competence as a collection of discrete traits or sub-competencies that are added together without considering possible interrelations or dynamic interactions between them. In such models, intercultural competence is treated as the sum of individual abilities, attitudes, and knowledge elements. This approach has been particularly influential in early research and practice, where the primary goal was to identify key characteristics that enable effective and appropriate interaction in intercultural settings. Although they vary in content, list models typically provide inventories of traits that are assumed to contribute to intercultural success, often serving as checklists for selection procedures, training needs analyses, or self-assessments.

One of the earliest and most cited examples of a list model is Ruben's (1976) framework of communication competence for intercultural adaptation. Ruben identified seven components that he considered essential for successful intercultural communication: display of respect, interaction posture, orientation to knowledge, empathy, self-oriented role behavior, interaction management, and tolerance for ambiguity. These elements reflect a strong emphasis on observable behaviors and interpersonal dynamics, highlighting the practical relevance of communication skills in intercultural contexts. Ruben's model emerged from the field of intercultural communication studies and was designed to describe the skills that facilitate adaptation in culturally unfamiliar environments, without offering a theoretical explanation of how these components relate to one another.

Another example of a list model is provided by Gaitanides (2003), who distinguishes between two broad domains of intercultural competence: intercultural action competence and intercultural cognitive competence. The former includes traits such as empathy, role distance, ambiguity tolerance, and communicative competence, capacities that enable individuals to act appropriately in intercultural situations. The latter encompasses culturally relevant knowledge domains, such as foreign languages, personal experiences abroad, knowledge of cultural standards, collective identity issues in immigration societies, as well as historical and socio-political backgrounds of migration. While this distinction acknowledges different facets of intercultural competence, it does not specify functional relationships between them. Consequently, despite its thematic grouping, the model remains structurally flat and is best understood as a list of components that may contribute to intercultural effectiveness, especially in the context of integration, education, or migration work.

List models are often based on surveys of people with experience abroad, e.g., international students or expatriates (Bolten, 2007). Due to their simple structure, they are easy to understand, but do not reflect the complexity of the research subject. In contrast to list models, structural models aim to capture the complexity of intercultural competence by organizing its components into superordinate dimensions and considering their functional interrelations. Rather than viewing individual traits or abilities as isolated, structural models conceptualize intercultural competence as a system of interconnected cognitive, affective, and behavioral elements. These models are often grounded in psychological theories of human functioning, particularly those emphasizing the interplay between attitudes, knowledge, motivation, and behavior. By providing a more integrated view, structural models attempt to explain not only *what* competencies are involved in intercultural effectiveness, but also how these competencies interact and evolve in specific contexts. This makes them particularly relevant for research aiming to understand the mechanisms underlying successful intercultural adaptation, as well as for the development of targeted interventions and training programs.

A well-known structural model of intercultural competence was proposed by Müller and Gelbrich (2004), who explicitly linked intercultural competence to the challenges of living and working abroad. Their model distinguishes between three core dimensions, affective, cognitive, and conative, which together reflect the emotional, mental, and behavioral capacities required for successful intercultural interaction. The affective component includes attitudes and motivations such as low ethnocentrism, openness, impartiality, and empathy. The cognitive dimension involves awareness and understanding of cultural norms, conventions, and rules that shape thought and behavior, including elements like cultural awareness, self-awareness, self-confidence, and the ability to form realistic expectations. The conative component refers to skills and values that support effective behavior in foreign cultural contexts, such as respect, flexibility, language competence, communication skills, and tolerance of ambiguity. In addition to these internal components, the model introduces two external criteria to evaluate intercultural success: *effectiveness* (measured through social and professional adjustment, satisfaction, and the desire to remain abroad) and *appropriateness* (the degree to which individuals follow local cultural rules and are sensitive to the emotional responses of hosts). This comprehensive framework thus not only classifies sub-competencies but also relates them to concrete outcomes of intercultural encounters.

Building on the cognitive—affective—conative structure, Dauner (2011) introduced a more dynamic perspective with his two-level model of intercultural competence. In this model, the first level comprises the familiar triad of cognitive (knowledge and awareness), affective (attitudes and openness), and behavioral (skills and action) components. What sets Dauner's approach apart is the inclusion of a second, regulating *meta-level*, which encompasses the ability to coordinate, adapt, and further develop these components in response to varying intercultural demands. This meta-level functions as a flexible control mechanism that enables individuals to modify their competencies depending on situational factors and cultural context. Rather than seeing competence as a static set of traits, the model emphasizes developmental and self-regulatory processes. By highlighting the importance of reflection, integration, and adaptation, Dauner's model offers a valuable conceptual bridge between structural and developmental approaches and is especially relevant for contexts where intercultural competence is not only required, but also cultivated over time.

Although the differentiation between various sub-dimensions of intercultural competence initially appears to be useful, structural models do not usually take sufficient account of the interdependence of these sub-dimensions. Training programs based on these models therefore often divide exercises into sub-dimensions artificially, e.g. exercises for affective, cognitive and conative aspects of competence, without establishing a reciprocal relationship between the different methods (Bolten, 2007).

Leung et al. (2014) proposed an influential integrative framework that has shaped much of the recent debate on intercultural competence. Developed on the basis of their extensive review of the literature, the framework sought to bring conceptual clarity to a fragmented field. Its guiding idea is that intercultural competence should not be reduced to isolated skills or traits, but rather conceived as the interplay of three clusters, traits, attitudes, and capabilities, that together enable intercultural effectiveness. Although Leung et al.'s (2014) framework has been highly influential in clarifying the components of intercultural competence, empirical research has produced mixed support for its predictive pathways. Several recent studies consistently demonstrate that capabilities, often operationalized through cultural intelligence or performance-based measures, are the most robust predictors of intercultural effectiveness. For example, Schelfhout et al. (2022) found that motivational and cognitive dimensions of cultural intelligence strongly predicted self-efficacy in intercultural encounters, while the influence of traits and attitudes was largely indirect. In a follow-up validation study, Schelfhout et al. (2024) confirmed that intercultural attitudes predicted capabilities, which in turn predicted effectiveness, but the direct link between attitudes and effectiveness was weak. Simulation-based studies such as the Direct Intercultural Effectiveness Simulation (Schelfhout and Derous, 2023) similarly highlight capabilities as the central explanatory factor, while traits and attitudes contribute only partially. Additional evidence from experimental settings supports this pattern: Vandecasteele et al. (2024) showed that empathy-related traits predicted aspects of physician—patient communication quality, though not consistently across tasks, whereas Schwarzenthal et al. (2020) demonstrated that classroom-level attitudes toward diversity influenced competence indirectly through their effects on cultural intelligence and situational judgment performance. Taken together, these findings suggest that traits and attitudes matter, but mainly as antecedents or enabling conditions rather than as definitional elements of intercultural competence. Positioning capabilities as the core of interculturally competent behavior thus seems more adequate, while recognizing the developmental role of dispositions and orientations in shaping how competence is acquired and enacted.

#### 3.1.2 Development models

Development models emphasize how intercultural competence evolves over time, framing it as a learning process that transforms an individual from being culturally unaware to interculturally competent. The most influential example is the Development Model of Intercultural Sensitivity (DMIS; Bennett, 1986; Hammer, 2012), which conceptualizes competence as trainable. The model outlines a five-stage continuum where higher levels reflect greater perceptual complexity and cultural sensitivity. Those with more cognitive complexity can better recognize and interpret cultural differences.

Originally comprising six stages (Bennett, 2017), the updated model includes denial, defense, minimization, acceptance, adaptation, and integration, ranging from a monocultural to an intercultural mindset. Development occurs through exposure to diverse social experiences that help individuals perceive and reflect on cultural complexity (Bennett, 1986; Hammer, 2012). Individuals at the denial stage ignore cultural differences, while those at the integration stage view such differences as part of their identity.

While intercultural sensitivity is a key component (Deardorff, 2006), it is not sufficient alone to define intercultural competence Genkova, (2019). The DMIS contributes an essential constructivist insight: the perception of cultural difference is shaped through experience and reflection.

(Genkova 2019) further distinguishes between models based on their understanding of competence as a discrete skill, like many structural model do (Graf and Mertesacker, 2010), or as a meta-competence, defined as the ability to act effectively in diverse cultural contexts through non-judgmental awareness and adaptive behaviour (Leung et al., 2014).

Some models see intercultural behaviour as stemming from personality or intelligence, despite differing from traditional definitions of competence. Still, these models are empirically supported (van der Zee and van Oudenhoven, 2001, 2013; van Dyne et al., 2009). As (Genkova 2019) emphasizes, the underlying model influences how competence is measured and interpreted. Especially in intercultural contexts, defining the construct and addressing methodological challenges are essential to developing valid assessment tools.

## 4 Measuring intercultural competence

### 4.1 Measurement in psychology

Measurement, understood as the assignment of numbers to individual characteristics following defined rules, enables the depiction of empirical relationships (Renner et al., 2012). Measuring intercultural competence therefore involves establishing rules to translate specific traits into numerical values. The quality of such a method is judged by its objectivity (results independent of the examiner), reliability (low or consistent measurement error), and validity (measuring what it intends to measure). Validity includes construct and criterion validity. For instance, a measure of intercultural competence with high criterion validity should correlate with cultural adaptation, academic or professional success, and well-being abroad.

Another key criterion is equivalence, meaning the method must meet quality standards across different groups, such as cultures. This includes functional equivalence (behaviours solve similar problems across cultures), conceptual equivalence (shared underlying constructs), and metric equivalence (consistent psychometric properties). To ensure this, tests must demonstrate scale equivalence and survey equivalence, that the same characteristics yield comparable responses across cultures (Genkova, 2019).

Currently, there is both a proliferation of definitions and an overwhelming variety of tools for measuring intercultural competence, including questionnaires, performance tests, interviews, observations, and diaries (Deardorff, 2009). Evaluating these instruments requires substantial methodological expertise. To guide the development of measurement instruments, Genkova (2019) proposes six criteria:

1. Desired effects: What does intercultural competence contribute to? Which outcomes are particularly desirable, or what is the focus?
2. Culture-specific or general competence: Should characteristics and skills be considered that are relevant to a specific cultural context, or is that construct to be measured as contributing to effective and appropriate behavior in any culture? Most existing measurement instruments focus on general intercultural competence without adequately addressing this requirement.
3. Which characteristics and skills (KSAO: knowledge, skills, abilities, other) are necessary to actually achieve the identified consequences?
4. What methods can be used to measure these characteristics and skills? If necessary, identification of suitable items.
5. Validity of content: Do the measurement methods reflect the content of the constructs formulated in steps one to three?
6. Does the instrument actually measure the intended construct? This step refers to the statistical examination of validity, in particular construct and criterion validity as well as external validity. Predictive validity is also considered less frequently.

This article has thus far focussed on the foundational understanding, effects, and structure of intercultural competence, which are addressed in the first three criteria. The following sections will build on these foundations and evaluate current and emerging instruments.

### 4.2 Measurement instruments

Instruments for measuring intercultural competence, or related constructs, are generally classified as direct methods (e.g., observation, performance tasks) or indirect methods (e.g., surveys, inventories) (Deardorff, 2011). Leung et al. (2014) further distinguish three main approaches: self-report, informant-based, and performance-based measurements, each with specific limitations.

Self-report is the most commonly used method. It involves individuals rating their own traits, such as in the Cultural Intelligence Scale (CQS; van Dyne et al., 2009), which assesses four sub-dimensions on a 7-point Likert scale. These measures reflect self-perception and are vulnerable to social desirability bias, an issue still under-researched (Leung et al., 2014; Lustig et al., 2009). Additionally, self-reports tend to capture competence statically but have been criticized for failing to represent its processual nature (Gabrenya et al., 2013). As Wolff (2017) notes, such instruments often stem from list or structural models, which limits their ability to assess dynamic intercultural processes. Informant-based assessments rely on observer evaluations, but their accuracy is limited by observers' perception and differing observational contexts. Consequently, this method is rarely applied in practice.

Performance-based methods assess observable behavior in intercultural scenarios or descriptions thereof to infer future potential. The most widely used approach in this category are Situational Judgment Tests (SJTs), which present participants with a scenario and ask them to select or evaluate a response option. Their appeal lies in their attempt to simulate real-life intercultural challenges and thereby move closer to applied judgment than abstract self-reports. Yet, as Leung et al. (2014) already observed, such measures face inherent limitations. The definition of what counts as “competent” behaviour is not universal but context-dependent, which makes SJTs vulnerable to cultural bias. What is perceived as appropriate in one culture may be judged differently in another. Consequently, SJTs often measure the ability to recognize culturally normative behaviour rather than intercultural competence in a generalizable sense. Differences in test familiarity, scenario interpretation, and underlying cultural norms further complicate their equivalence across groups.

Recent debates have added several layers to this critique. One major issue concerns scoring practices. Some SJTs rely on expert ratings of the most effective response, while others use consensus-based scoring. Both have drawbacks: expert scoring risks privileging dominant cultural viewpoints, whereas consensus scoring may reproduce conformity rather than genuine intercultural sensitivity (Rockstuhl et al., 2015; Chen, 2025). Another discussion revolves around the balance between response judgment and situational judgment. Traditional SJTs emphasize the choice of responses, but research shows that assessing how individuals interpret the situation itself offers incremental predictive validity for intercultural effectiveness (Rockstuhl et al., 2015). Scholars have also questioned the construct clarity of SJTs. Because they often capture a mixture of cultural knowledge, social judgment, cognitive ability, and personality, it remains ambiguous what exactly they measure (Leung et al., 2014; Schelfhout et al., 2022).

To address these challenges, recent innovations have emerged. The Brief Test of Intercultural Judgment (BTIJ), for example, provides a concise and validated format tailored to education professionals, demonstrating positive associations with both intercultural knowledge and performance (Starčević et al., 2023). The Direct Intercultural Effectiveness Simulation (DIES) represents another step forward by integrating Leung et al.'s (2014) framework with Implicit Trait Policy theory. This approach allows researchers to examine how traits, attitudes, and capabilities interact in shaping intercultural decision-making (Schelfhout and Derous 2023). Together, these developments illustrate both the promise and the limits of SJTs. Each method has strengths and weaknesses, highlighting the need for multi-method strategies. Leung et al. (2014) advocate for broader methodological integration. Deardorff (2011) and Genkova (2019) recommend grounding measurement in clearly defined, context-specific constructs, with goals, indicators, and methods aligned accordingly. Genkova further emphasizes multi-method (qualitative and quantitative) and multi-perspective (e.g., target group-informed) approaches. However, most existing tools only partially meet these standards.

## 5 Available instruments for measuring intercultural competence

Only a small proportion of existing instruments for assessing intercultural competence have undergone thorough empirical validation. Gabrenya et al. (2012), expanding on Matsumoto and Hwang (2013), evaluated 34 instruments and found adequate criterion validity in just 14. Both studies emphasize criterion validity as the key standard for evaluating such tools. Based on their findings and those of Wolff (2017), twelve instruments with available psychometric data are highlighted in Table 1.

**Table 1:** Questionnaires and Tests of Intercultural Competence.

**Measurement procedure**	**Dimensions/Items**	**Construct understanding**	**Quality criteria**
CCAI: Cross-Cultural Adaptability Inventory (Kelley and Meyers, 1995)	4/50 Emotional resilience, flexibility/openness, perceptual abilities, personal autonomy	Intercultural personality traits	α = 0.68-0.90 Content validity: Appropriate construct validity: Factor structure not supported; no statement possible
CCSS: Cross-Cultural Sensitivity Scale (Pruegger and Rogers, 1993)	1/24 Cultural knowledge, attitudes, convictions and lifestyles	World views, intercultural abilities	α = 0.80-0.87 Content validity: appropriate construct validity: A hardly possible statement criterion validity: a hardly possible statement
CQS: Cultural Intelligence Scale (van Dyne et al., 2009)	4/20 Metacognition,cognition,motivation,behavior	Intercultural skills	α = 0.77-0.84 Content validity: appropriate Construct validity: convincing Criterion validity: convincing
ICAPS: Intercultural Adjustment Potential Scale (Matsumoto et al., 2001)	4/55 Emotion regulation, openness, flexibility, critical thinking	Intercultural personality traits	Content validity: appropriate construct validity: sufficient criterion validity: convincing
ICC: Intercultural Communication Competence (Arasaratnam et al., 2010)	3/10 cognitive, affective, behavioral	Intercultural personality traits intercultural skills	α = 0.77 Content validity: questionable construct validity: factor structure questionable, a statement hardly possible but rather existing criterion validity: no statement possible
ICSI: Intercultural Sensitivity Inventory (Bhawuk and Brislin, 1992)	4/46 Individualism, collectivism, open-mindedness, flexibility	Intercultural attitudes, world views, intercultural skills	α = 0.82-0.84 Content validity: appropriate construct validity: a statement hardly possible,tends to being available criterion validity: a statement hardly possible, tends to be available
IDI: Intercultural Development Inventory (Hammer, 2012)	7/50 Refusal, defense, reversal, minimization, acceptance, adjustment, cultural disentanglement	Intercultural attitudes, world views	α = 0.80-0.85 content validity: appropriate construct validity: inconsistent criterion validity: a statement hardly possible, inconsistent
ISS: Intercultural Sensitivity Scale (Chen and Starosta, 2000)	5/24 Engagement, respect for cultural differences, interaction trust, interaction enjoyment, interaction attention	Intercultural personality traits, intercultural skills	Content validity: appropriate Construct validity: a statement hardly possible Criterion validity: a statement hardly possible, tends to be available
MPQ: Multicultural Personality Questionnaire (van der Zee and van Oudenhoven, 2001)	5/78 Cultural empathy, openness, emotional stability, social initiative, flexibility	Intercultural personality traits	α = 0.95 Content validity: Questionable Construct validity: convincing Criterion validity: convincing
MPQ-SF: Multicultural Personality Questionnaire – Short Form (van der Zee et al., 2013)	5/40 Cultural empathy, openness, emotional stability, social initiative, flexibility	Intercultural personality traits	Content validity: questionable Construct validity: convincing Criterion validity: convincing
TMIK: Test for the measurement of intercultural competence (Schnabel et al., 2014)	16/75 + 16 SJTs Readiness for the use of a foreign language, empathy in communication, targeted collection of information, building of trusting relationships, integration in groups, perspective change in communication, flexibility in communication, Clarity in communication, readiness to learn, mediation of different interests, building of a professional network, strategic problem-solving, establish and maintain contacts, goal orientation, enable productive collaboration, reflection on one's own culture, awareness of one's own culture	Intercultural skills	α = 0.69−0.90 Content validity: appropriate construct validity: appropriate criterion validity: inconsistent equivalence: partially satisfied
TMIK-S: Test for the measurement of intercultural competence Short Form (Schnabel et al., 2015)	Empathy in communication, targeted collection of information, establishing and maintaining contacts, goal orientation, mediation of different interests, reflection on one's own culture	Intercultural skills	α = 0.65 -0.86 Content validity: appropriate Construct validity: appropriate Criterion validity: inconsistent, equivalence: partially satisfied
DIES: Direct Intercultural Effectiveness Simulation (Schelfhout and Derous, 2023)	Multimedia format (text and visuals) measuring Implicit Trait Policies (ITPs) as latent intercultural effectiveness	Intercultural skills	Reliability: α > 0.80 Validity: Construct validity supported by SEM: intercultural traits, attitudes, and CQ significantly predicted DIES scores. Criterion validity shown by associations with intensity of intercultural contact and related outcomes. Incremental validity beyond personality and general intelligence demonstrated. Equivalence: not yet tested

This is an edited and extended version of the research of (Wolff 2017) as well as (Matsumoto and Hwang 2013). The column construct understanding describes on which understanding of Intercultural Competence according to (Leung et al. 2014) is the respective instrument based on.

Of these, only three meet quality standards: the Cultural Intelligence Scale (CQS), the Multicultural Personality Questionnaire (MPQ), and the Test for Measuring Intercultural Competence (TMIK). Despite known conceptual and methodological limitations, these tools are considered the most robust currently available (Genkova, 2019).

### 5.1 Cultural Intelligence Scale (CQS)

Cultural Intelligence (CQ) was introduced by Earley and Ang (2003) as a multidimensional construct to explain individual differences in adapting to culturally diverse settings. They posited that successful intercultural adaptation depends on specific individual capabilities beyond general cognitive ability. CQ is conceptualized as comprising cognitive, metacognitive, motivational, and behavioral dimensions, and intersects conceptually with emotional and social intelligence (Earley and Ang, 2003). Although CQ has been linked in the literature to Gardner (1985) theory of multiple intelligences, the original framework by Earley and Ang does not explicitly draw on it.

Initially, Earley and Ang identified three dimensions of CQ, but this framework was later expanded by van Dyne et al. (2009) to encompass four core dimensions: cognitive, metacognitive, motivational, and behavioural. The cognitive dimension refers to knowledge of cultural systems, norms, and practices. The metacognitive dimension involves cultural awareness and reflective thinking, enabling individuals to anticipate, recognize, and adjust to cultural differences in real time. The motivational dimension captures an individual's intrinsic interest and confidence in functioning in diverse cultural settings, which facilitates sustained engagement. Finally, the behavioural dimension reflects the capacity to adapt one's verbal and non-verbal actions appropriately across cultural contexts (Ang and Van Dyne, 2008).

To operationalize the model, van Dyne et al. (2009) developed the Cultural Intelligence Scale (CQS), a 20-item instrument rated on a seven-point Likert scale. Despite being translated into numerous languages, the CQS is still widely used in its original English version due to its simplicity and cross-cultural relevance (Rockstuhl and van Dyne, 2018). The CQS has been validated across diverse samples, including expatriates in Singapore and the U.S. (van Dyne et al., 2009), Filipino expatriates in Taiwan (Chen et al., 2011), employees in Taiwanese firms (Lee and Sukoco, 2010), Korean university students (Moon, 2010), and cadets at a Swiss military academy (Rockstuhl et al., 2012). These applications confirm the instrument's robust psychometric properties and broad utility across both Western and East Asian cultural contexts.

A German-language adaptation of the CQS was validated by Greischel et al. (2021), demonstrating its relevance for both expatriates and local professionals in German-speaking settings. However, the scale's applicability in other European regions, particularly Eastern and Southeastern Europe, remains underexplored, leaving a gap in empirical validation for these contexts.

### 5.2 Multicultural Personality Questionnaire (MPQ)

The Cultural Intelligence (CQ) model emphasizes cognitive, affective, and behavioral components relevant to intercultural situations. However, alternative approaches examine more distal predictors of intercultural competence, particularly stable individual differences such as personality traits. Tett and Guterman (2000) defined personality as the sum of intra-individually stable and inter-individually unique latent behavioral tendencies that manifest depending on the situational context. From this perspective, it is plausible that effective behavior in intercultural settings can be predicted by personality traits specifically relevant to navigating cultural diversity.

Drawing on the Big Five model, van der Zee and van Oudenhoven (2000) identified five traits uniquely associated with multicultural effectiveness:

1. Cultural empathy, the capacity to understand and relate to the thoughts, emotions, and behaviors of individuals from different cultural backgrounds;
2. Open-mindedness, an unbiased, receptive attitude toward unfamiliar cultural norms and experiences;
3. Social initiative, a proactive tendency to engage socially in unfamiliar contexts;
4. Emotional stability, the ability to remain composed under stress and uncertainty;
5. Flexibility, the capacity to view novel situations as opportunities for growth and to adapt accordingly (van der Zee et al., 2013).

Theoretically, individuals high in emotional stability and flexibility are better equipped to manage the psychological stress associated with cultural novelty and ambiguity. Those scoring high in social initiative and open-mindedness tend to approach intercultural challenges with less anxiety and greater engagement. Meanwhile, individuals with high cultural empathy are more adept at recognizing and responding to diverse cultural norms, thereby reducing interpersonal friction and fostering more constructive interactions (van der Zee and van Oudenhoven, 2013).

To assess these traits, van der Zee and van Oudenhoven (2000) developed the Multicultural Personality Questionnaire (MPQ), originally comprising 90 items rated on a five-point Likert scale. A validated short version with 40 items was later introduced (van der Zee and van Oudenhoven, 2013). The MPQ has demonstrated strong psychometric properties, including internal consistency, test-retest reliability, convergent and discriminant validity, and partial scalar and metric invariance across cultural groups (Genkova et al., 2021; van der Zee et al., 2013; Wöhrle et al., 2015).

Although personality traits are considered relatively stable, their expression may vary across cultures due to differences in social norms, interpretive schemas, and situational expectations (McCrae and Terracciano, 2005). Nevertheless, the MPQ has been widely applied and validated across numerous cultural contexts. Originally developed in the Netherlands, it has been used in Spain (Bobowik et al., 2011), Belgium (van der Zee and van Oudenhoven, 2013), and the United States (Houtz et al., 2010), among others. Przytula et al. (2024) found significantly higher MPQ scores among students who participated in short-term international training compared to a non-training control group, suggesting that intercultural experiences may lead to measurable changes in MPQ dimensions. The scale has also been administered to employees of Dutch multinational firms (Korzilius et al., 2011), Western expatriates in Taiwan (van der Zee et al., 2013), and Canadian expatriates (Simkhovych, 2009). Across these contexts, cultural empathy, flexibility, and emotional stability have consistently emerged as key predictors of successful intercultural adaptation.

However, despite its widespread application, the MPQ has predominantly been validated in Western and Far Eastern settings, with limited empirical evidence available for other regions, particularly Eastern and Southeastern Europe.

Despite the considerable effort put into researching intercultural competence, many studies lack considerations of reliability and validity as well as a plausible definition of the concept of competence (Schnabel et al., 2015). Based on the understanding of competence and intelligence presented above, the CQS, for example, cannot be used to measure intelligence, as no innate abilities are assessed. The approaches of intercultural competence and intercultural intelligence are not differentiated from one another, which results in validity deficits. Both the CQS and the MPQ are based on constructs, such as personality traits and cognitive-motivational dispositions, that are typically considered relatively stable, even though their expression may change with experience or targeted interventions. Less common models are usually not tested at all for the generalizability of the assumed factor structure across cultures (Rockstuhl and van Dyne, 2018). Contradictory results were found for the MPQ and the CQS, which reinforces doubts whether the respective instruments measure generalizable competence (Genkova et al., 2025; Rockstuhl and van Dyne, 2018). (Rockstuhl and van Dyne 2018) also show a bi-factorial structure of the CQS (variance in the items is explained by both a domain-specific and a superordinate factor). This could indicate that the variance in the items is only partially explained by a general intercultural intelligence factor (which may reflect cognitive performance) and the rest is explained by specific abilities.

### 5.3 Test for Measuring Intercultural Competence (TMIK)

The TMIK (Test zur Messung interkultureller Kompetenz), developed by Schnabel et al. (2014), combines a self-report scale with SJTs to assess intercultural competence across 17 dimensions. It builds on an extensive review of existing models and is designed to capture both attitudinal and behavioral components of intercultural competence.

The psychometric validation of the TMIK included indicators such as time spent abroad, participation in intercultural training, and language proficiency. Studies report that TMIK correlates well with external markers of intercultural experience (Schnabel et al., 2014, 2015). Measurement invariance testing between German and Brazilian samples showed scalar invariance, but significant latent mean differences across multiple dimensions indicate that the TMIC-S does not perform uniformly across cultures. (Schnabel et al., 2015). The study also provides evidence for the incremental validity of its SJT component over the self-report scale. However, separating measured competence from underlying traits such as personality or general intelligence remains challenging and is a broader concern in SJT-based competence assessment (Genkova, 2019).

As an intermediate conclusion, it can be stated that intercultural competence is not validly measured by any of the existing measurement instruments. The MPQ and the CQS have clear limitations, both conceptually and in terms of differentiation from the constructs of personality or intelligence. Empirical results for the scales are contradictory and indicate problems with criterion validity. Despite some improvements, the results of the TMIK differ between the German and Brazilian samples. No other results on equivalence are available. The interactions between intercultural competence and cultural distance are also very heterogeneous.

The major problems in measuring intercultural competence in different cultures are overall indicators that intercultural competence itself is a culture-bound construct. It can therefore be assumed that intercultural competence is at least partly culture-specific (Bolten, 2016; Genkova, 2019). The measurement and promotion of intercultural competence must be adapted to the respective cultural context. The only known method that takes these challenges and conclusions into account is the Cult-Euro-1 test, which is presented below.

### 5.4 Introducing Cult-Euro-1: test for general and culture-specific intercultural competence

The Cult Euro 1 Test is based on the understanding of intercultural competence general cognitive and context-specific performance disposition that is a necessary condition for the successful and adequate interaction of individuals from different cultural contexts, as presented in this paper. A central insight emerging from the theoretical and empirical literature on intercultural competence is that effective interaction in culturally diverse contexts requires the interplay of both general (context-independent) and culture-specific (context-dependent) components (Thomas, 2002; Deardorff, 2009; Genkova, 2019). General intercultural competence refers to cross-situational psychological dispositions and skills that facilitate orientation, communication, and adaptation across a wide range of cultural contexts. In contrast, culture-specific competence captures the abilities that are essential for effective interaction within a particular cultural setting.

While the expression and relevance of intercultural competence are likely to be shaped by specific cultural norms and institutions (Thomas, 1993; Bolten, 2016), there is broad agreement and empirical support that certain underlying abilities support adaptation across contexts (Leung et al., 2014; Schnabel et al., 2014). Such abilities are conceptualized as general intercultural competence and are reflected in validated measurement instruments such as the Multicultural Personality Questionnaire (MPQ; van der Zee and van Oudenhoven, 2000) and the Cultural Intelligence Scale (CQS; van Dyne et al., 2009). These tools focus on psychological capacities that predict intercultural effectiveness across varied environments, though they do not systematically account for the specific sociocultural knowledge required in any given national or institutional setting.

Culture-specific intercultural competence, by contrast, involves an understanding of particular behavioral scripts, institutional practices, and communication norms that are distinctive to a cultural group. This includes, for example, familiarity with forms of address, patterns of formality, local expectations around punctuality or emotional expressiveness, and culturally normative interpretations of behavior. An example for previous approaches to address culture specific competence are cultural assimilators. Developed primarily for training purposes, these instruments present users with culture-specific scenarios and evaluate their ability to select appropriate behavioral responses (Bhawuk and Brislin, 1992). Although effective for cultural orientation, such tools typically lack a foundation in competence research and empirical validation, limiting their utility for psychological assessment (Gabrenya et al., 2012). Critical researchers also emphasize that presenting stereotypes of a certain cultural context is not the same as gaining competence and may even be detrimental, since it may reinforce stereotypes (Borghetti, 2017; Holliday and Macdonald, 2020). Thus it is important to emphasize that culture specific intercultural competence does not refer to assimilation but to the ability to interact successfully with members of a certain cultural group.

#### 5.4.1 Development of the Cult-Euro-1 test

To establish an empirically grounded framework for the development of Cult-Euro-1, an extensive qualitative study was conducted with the aim of identifying core dimensions of both general and culture-specific intercultural competence. This inductive approach was chosen to overcome the limitations of existing instruments, which often rely heavily on theoretical assumptions or lack contextual sensitivity. The qualitative study served a dual purpose: first, to validate the conceptual distinction between general and culture-bound competence domains; second, to derive concrete situational content and psychological attributes that could inform item construction. The study was grounded in a theory-led interview framework and followed a bottom-up logic to capture the subjective perspectives and lived experiences of individuals in intercultural situations within the German context. The analytical strategy combined qualitative content analysis (Mayring, 2019) with the Critical Incident Technique to identify behaviorally anchored challenges and responses relevant to intercultural encounters. This methodological design ensured that the resulting competence dimensions reflected both abstract cognitive-affective traits and observable situational demands in a culturally meaningful way.

A total of two hundred and fifty individuals were interviewed, selected to represent diverse social roles and degrees of familiarity with the German cultural context. The sample included international students (*n* = 57), students with a migration background who had completed their schooling in Germany (*n* = 38), students without a migration background (*n* = 24), expatriates currently working in Germany (*n* = 6), refugees *(n* = 40), unemployed individuals (*n* = 26), professionals (*n* = 40), and domain experts in intercultural competence (*n* = 20). The interviews were analyzed using qualitative content analysis in accordance with the method developed by Mayring (2019). This involved a structured, multi-stage process of paraphrasing participant responses, generalizing their content, and categorizing them into higher-order thematic clusters. The goal was to reduce the data systematically while preserving contextual meaning relevant to intercultural competence. In addition to this content-analytic procedure, the Critical Incident Technique was employed to identify key situations and behavioral patterns that respondents perceived as particularly challenging or illustrative of competent intercultural behavior.

The combined analysis of interview data led to the identification of 106 distinct content categories, which were subsequently grouped into 58 dimensions of general intercultural competence and 48 dimensions of German culture-specific competence. General competence dimensions included personal dispositions such as openness, flexibility, and emotional stability, as well as cognitive-affective skills like empathy, perspective-taking, and reflexivity. These were complemented by attitudes toward cultural diversity, including respect, tolerance of ambiguity, and egalitarian values. In contrast, the culture-specific categories reflected concrete behavioral expectations and sociocultural knowledge relevant to navigating German societal norms. These included punctuality, formality in communication, institutional practices (e.g., rules, laws, educational systems), gender norms, and culturally typical interaction styles. The distinction between general and specific components was empirically grounded: while general traits were reported as broadly helpful across contexts, culture-specific elements were described as essential within German society, but potentially counterproductive elsewhere. This conceptual differentiation formed the basis for the modular structure of the test.

The resulting category system provided a comprehensive and empirically grounded framework for the operationalization of intercultural competence in Cult-Euro-1. Drawing on established models of competence as situational action potential (Erpenbeck, 2012), the identified categories were not treated as static traits but as context-sensitive dispositions that manifest in response to specific intercultural demands. Each cluster captured a distinct aspect of competence that could be translated into measurable constructs through self-assessment items and situational judgment scenarios. This structure allowed for the systematic differentiation of competence facets across cognitive, affective, and behavioral domains, and aligned with theoretical perspectives emphasizing the dynamic, interactional nature of intercultural competence (e.g., Deardorff, 2009; Thomas, 2002).

To evaluate the initial item pool and examine its psychometric quality, a quantitative pretest was conducted between February and April 2022. The study aimed to assess the internal structure of the test, investigate convergent validity with established intercultural competence instruments, and identify subscales requiring refinement. A total of two hundred and twenty seven complete datasets were analyzed after the exclusion of incomplete responses and statistical outliers. Participants were recruited via social media and student assistant networks and represented a heterogeneous group in terms of age, gender, migration background, and educational attainment. The survey was administered online via LimeSurvey, with a minimum language requirement of B2 in German. Informed consent and data protection procedures followed APA ethical standards. To validate the new instrument, participants also completed established measures including the TMIK-S, the Multicultural Personality Questionnaire (MPQ), the Mini-Cultural Intelligence Scale (CQS), the Scale of Ethnocultural Empathy (SEE), and the Sociocultural Adaptation Scale (SCAS). Table 2 presents the bivariate correlations with established measurement tools. The results provided initial support for the proposed distinction between general and culture-specific intercultural competence and confirmed expected correlations with external criteria, indicating good convergent validity. After reviewing the itempool to improve unidimensionality and reliability, the questionnaire for the main validation study was launched.

**Table 2:** Bivariate correlations of the subscales of general intercultural competence with established scales of intercultural competence.

**N**.	**Scale**	**TMIKS**	**MPQ**	**SEE**	**CQS**	**SCAS**
1	Cultural learning	0.66^**^	0.55^**^	0.64^**^	0.58^**^	0.46^**^
2	Freedom from prejudice	0.36^**^	0.39^**^	0.67^**^	0.33^**^	0.21^**^
3	Agreeableness	0.60^**^	0.64^**^	0.64^**^	0.55^**^	0.39^**^
4	Empathy	0.43^**^	0.55^**^	0.44^**^	0.38^**^	0.29^**^
5	Communication	0.54^**^	0.53^**^	0.67^**^	0.48^**^	0.27^**^
6	Acceptance	0.38^**^	0.40^**^	0.63^**^	0.43^**^	0.34^**^
7	Flexibility	0.44^**^	0.43^**^	0.46^**^	0.47^**^	0.36^**^
8	Reflection	0.51^**^	0.38^**^	0.57^**^	0.44^**^	0.23^**^
9	Tolerance of ambiguity	0.04	0.20^*^	0.26^*^	0.03	0.06
10	Self-confidence	0.56^**^	0.63^**^	0.41^**^	0.58^**^	0.57^**^
11	Extraversion	0.54^**^	0.68^**^	0.43^**^	0.45^**^	0.41^**^
12	Work	0.31^**^	0.23^**^	0.01^*^	0.28^**^	0.23^**^
13	Rule orientation	0.29^**^	0.25^**^	0.24^**^	0.28^**^	0.09^*^
14	Culture specific knowledge	0.62^**^	0.57^**^	0.33^**^	0.46^**^	0.38^**^
15	Communication	0.38^**^	0.49^**^	0.28^**^	0.35^**^	0.27^**^
16	Punctuality	0.31^**^	0.38^**^	0.18^**^	0.22^**^	0.20^**^
17	Gender competence	0.18^*^	0.19^*^	0.47^*^	0.29^*^	0.05

(^*^correlation is two-sided significant at the level of p < 0.05; ^**^ correlation is two-sided significant at the level of p < 0.001).

The main validation study of Cult-Euro-1 aimed to finalize the psychometric structure of the test, calibrate item parameters using item response theory (IRT), and establish reference norms across diverse population groups. A particular focus was placed on evaluating the factorial validity and reliability of the revised subscales, examining item functioning across different demographic segments, and preparing the test for standardized application in both research and practice. To achieve this, a stratified sample of *N* = 6,294 participants was recruited across thirteen predefined subgroups, representing key intersectional profiles of intercultural experience. These included German students, students with and without migration background, international students, and refugee students, as well as professionals and unemployed individuals with similar subgroup distinctions. Data collection was facilitated through online panels. To establish construct and criterion validity, participants completed the revised Cult-Euro-1 instrument alongside a comprehensive battery of related psychological measures. These included established scales assessing intergroup contact (Islam and Hewstone, 1993), emotional responses in intercultural contexts (Kauff et al., 2017), cultural stress (Thomson et al., 2006), loneliness (adapted from the IAB-BAMF-SOEP), and depression symptoms. The integration of these measures enabled a nuanced assessment of how intercultural competence relates to psychosocial adjustment and real-life intercultural experiences. From a psychometric standpoint, the revised item pool underwent rigorous statistical modeling. Confirmatory factor analyses were used to test unidimensionality and local independence of the subscales, resulting in the exclusion of items with weak factor loadings or residual correlations. Table 3 displays the model fit for the confirmatory factor analysis using WLMSV-estimators in lavaan. Tables 4, 5 present the correlations with the criteria of intercultural competence. Item Response Functions (IRFs) were evaluated both parametrically and non-parametrically, with problematic response patterns prompting either item removal or the collapsing of response categories. The final calibration of the test employed a two-parameter graded response model, allowing for variation in both item discrimination and difficulty parameters. Group comparisons and differential item functioning analyses further informed the selection of appropriate norm groups, and item parameters were standardized on the Stanine scale to enable interpretable scoring across populations.

**Table 3:** Fit indices of the unidimensionality of the subscales.

**N**.	**Scale**	**X^2^**	**df**	***p***	**CFI**	**TLI**	**RMSEA**
1	Cultural learning	1753.386	119	0	0.979	0.975	0.047
2	Freedom from prejudice	2079.241	135	0	0.985	0.983	0.048
3	Agreeableness	999.948	65	0	0.979	0.975	0.048
4	Empathy	322.978	27	0	0.986	0.981	0.042
5	Communication	253.731	27	0	0.990	0.987	0.037
6	Acceptance	399.404	27	0	0.979	0.973	0.047
7	Flexibility	582.92	35	0	0.968	0.959	0.050
8	Reflection	388.651	35	0	0.982	0.977	0.040
9	Tolerance of ambiguity	214.684	14	0	0.963	0.944	0.048
10	Self-confidence	229.673	9	0	0.982	0.971	0.063
11	Extraversion	38.944	5	0	0.996	0.993	0.033
12	Work	181.385	9	0	0.971	0.952	0.056
13	Rule orientation	82.577	9	0	0.988	0.98	0.037
14	Culture specific knowledge	97.126	14	0	0.991	0.987	0.031
15	Communication	181.639	9	0	0.971	0.951	0.056
16	Punctuality	175.78	5	0	0.982	0.965	0.075
17	Gender competence	301.338	20	0	0.992	0.989	0.048
18	Direct communication	37.669	5	0	0.995	0.991	0.033
19	Behavioral competence	1605.539	377	0	0.995	0.995	0.024

**Table 4:** Bivariate correlations of the subscales of general intercultural competence with criteria related to previous stays abroad.

	**General intercultural competence**										
**Kriterien/Skalen**	**1**	**2**	**3**	**4**	**5**	**6**	**7**	**8**	**9**	**10**	**11**
Stayabroad (y/n)	0.19^**^	0.06^**^	0.10^**^	0.1^**^	0.09^**^	0.07^**^	0.16^**^	0.12^**^	0.06^**^	0.21^**^	0.13^**^
Duration stay abroad	0.02	0.01	0.04	0.03	0.03	0.03	0.02	0.04	0	0	0.02
Contact frequency abroad	0.32^**^	0.02	0.32^**^	0.32^**^	0.29^**^	0.25^**^	0.33^**^	0.28^**^	−0.07^**^	0.33^**^	0.29^**^
Contact quality abroad	0.43^**^	0.18^**^	0.43^**^	0.37^**^	0.38^**^	0.40^**^	0.39^**^	0.36^**^	0.03	0.40^**^	0.35^**^
Negative contact abroad	−0.21^**^	−0.43^**^	−0.20^**^	−0.10^**^	−0.16^**^	−0.30^**^	−0.12^**^	−0.14^**^	−0.30^**^	−0.11^**^	−0.13^**^
Positive contact abroad	0.42^**^	0.24^**^	0.40^**^	0.33^**^	0.36^**^	0.40^**^	0.36^**^	0.36^**^	0.05^*^	0.35^**^	0.31^**^
Cultural stress abroad	−0.18^**^	−0.45^**^	−0.19^**^	−0.07^**^	−0.13^**^	−0.26^**^	−0.08^**^	−0.11^**^	−0.38^**^	−0.11^**^	−0.14^**^
Loneliness abroad	−0.11^**^	−0.36^**^	−0.12^**^	−0.04	−0.06^**^	−0.17^**^	−0.03	−0.03	−0.32^**^	−0.06^*^	−0.10^**^
Satisfaction with stay abroad	0.45^**^	0.08^**^	0.46^**^	0.41^**^	0.39^**^	0.38^**^	0.45^**^	0.38^**^	0.06^*^	0.44^**^	0.36^**^

(^*^correlation is two-sided significant at the level of p < 0.05; ^**^ correlation is two-sided significant at the level of p < 0.001).

**Table 5:** Bivariate correlations of the subscales of intercultural competence with criteria related to Germany.

	**General intercultural competence**											**German-specific intercultural competence**							
**Kriterien/Skalen**	**1**	**2**	**3**	**4**	**5**	**6**	**7**	**8**	**9**	**10**	**11**	**12**	**13**	**14**	**15**	**16**	**17**	**18**	**19**
Negative contact in GER	−0.07^*^	−0.22^**^	−0.14^**^	−0.09^**^	−0.08^**^	−0.16^**^	−0.11^**^	−0.04	−0.15^**^	−0.15^**^	−0.13^**^	−0.09^**^	−0.1^**^	−0.10^**^	−0.07^*^	−0.22^**^	−0.15^**^	−0.24^**^	−0.10^**^
Positive contact in GER	0.25^**^	0.18^**^	0.26^**^	0.25^**^	0.25^**^	0.17^**^	0.21^**^	0.19^**^	0.09^**^	0.23^**^	0.26^**^	0.04	0.12^**^	0.29^**^	0.20^**^	0.11^**^	0.17^**^	0.23^**^	0.02
Contact frequency in GER	0.19^**^	0.11^**^	0.17^**^	0.19^**^	0.17^**^	0.05	0.09^**^	0.10^**^	0.07^*^	0.19^**^	0.18^**^	−0.05	−0.01	0.29^**^	0.15^**^	0.12^**^	0.22^**^	0.26^**^	0.02
Contact quality in GER	0.22^**^	0.22^**^	0.24^**^	0.21^**^	0.22^**^	0.15^**^	0.16^**^	0.14^**^	0.12^**^	0.23^**^	0.23^**^	0.05	0.10^**^	0.34^**^	0.17^**^	0.23^**^	0.24^**^	0.36^**^	0.01
Negative contact in GER	−0.07^**^	−0.25^**^	−0.11^**^	−0.07^*^	−0.10^**^	−0.16^**^	−0.08^**^	−0.03	−0.14^**^	−0.09^**^	−0.11^**^	−0.08^**^	−0.12^**^	−0.09^**^	−0.08^**^	−0.24^**^	−0.15^**^	−0.27^**^	−0.12^**^
Positive contact in GER	0.29^**^	0.17^**^	0.27^**^	0.26^**^	0.26^**^	0.18^**^	0.21^**^	0.23^**^	0.07^*^	0.23^**^	0.24^**^	0.05^*^	0.12^**^	0.29^**^	0.20^**^	0.18^**^	0.22^**^	0.29^**^	0.01
Cultural stress in GER	−0.33^**^	−0.18^**^	−0.12^**^	-	−0.18^**^	−0.12^**^	−0.07^*^	−0.2^**^	−0.16^**^	−0.17^**^	−0.07^*^	−0.11^**^	−0.24^**^	−0.13^**^	−0.32^**^	−0.27^**^	−0.4^**^	−0.21^**^	−0.33^**^
Loneliness in GER	−0.03	−0.23^**^	−0.09^**^	−0.08^**^	−0.03	−0.08^**^	−0.06^*^	0.00	−0.19^**^	−0.12^**^	−0.14^**^	−0.04	−0.03	−0.13^**^	−0.04	−0.20^**^	−0.18^**^	−0.27^**^	−0.22^**^
Satisfaction with stay in GER	0.35^**^	0.22^**^	0.4^**^	0.33^**^	0.35^**^	0.32^**^	0.35^**^	0.29^**^	0.04	0.38^**^	0.34^**^	0.2^**^	0.25^**^	0.35^**^	0.28^**^	0.21^**^	0.18^**^	0.21^**^	0.14^**^
Depression	−0.11^**^	−0.24^**^	−0.16^**^	−0.15^**^	−0.08^**^	−0.15^**^	−0.12^**^	−0.06^*^	−0.17^**^	−0.22^**^	−0.21^**^	−0.09^**^	−0.08^**^	−0.11^**^	−0.05	−0.17^**^	−0.12^**^	−0.17^**^	−0.50^**^

(^*^correlation is two-sided significant at the level of p < 0.05; ^**^ correlation is two-sided significant at the level of p < 0.001).

The final Test is comprised of two modules that can be used independently. Table 6 contains the reliability indicators of the final test version, Table 7 displays the dimensions and example items. Cult-Euro-1 is distinguished by its theoretical foundation, strong empirical validation, and its status as the first standardized test of intercultural competence. Unlike earlier tools, it considers measurement relativity to a reference population and aims for a normal distribution of scores, as is typical for competencies rather than knowledge. The modular structure enables flexible use. The general module can be supplemented with specific cultural or occupational modules tailored to the context. Additional modules for other cultural or professional domains are planned.

**Table 6:** Classical test theory reliability indicators.

**Nr**.	**Scale**	**Cronbachs α**	***M***	***SD***	**Difficulty**
1	Cultural Learning	0.90	5.01	1.41	71.60
2	Freedom from Prejudice	0.92	4.31	1.69	61.59
3	Agreeableness	0.87	5.15	1.34	73.58
4	Empathy	0.83	4.92	1.33	7.27
5	Communication	0.84	5.11	1.33	72.99
6	Acceptance	0.77	5.12	1.45	73.19
7	Flexibility	0.79	4.88	1.37	69.70
8	Reflection	0.80	4.95	1.37	7.72
9	Tolerance of ambiguity	0.67	3.76	1.52	53.64
10	Self-confidence	0.82	4.82	1.41	68.80
11	Extraversion	0.79	4.69	1.58	66.99
12	Work	0.69	4.69	1.45	66.94
13	Rule orientation	0.70	5.18	1.41	73.93
14	Culture specific knowledge	0.72	5.03	1.46	71.88
15	Communication	0.70	4.97	1.41	7.93
16	Punctuality	0.78	5.06	1.72	72.28
17	Gender competence	0.87	4.57	1.84	65.29
18	Direct communication	0.75	4.46	1.64	63.73
19	Behavioral competence	0.89	0.46	0.49	6.58

**Table 7:** Final version of the test, example items.

**Nr**.	**Scale**	Items^*^	**Example Item**
1	Cultural learning	9	I enjoy getting to know other cultures.
2	Freedom from prejudice	10	I feel stressed by unfamiliar cultural rules.
3	Agreeableness	8	I can communicate well with people from other cultures.
4	Empathy	6	When conflicts arise, I try to form my own opinion.
5	Communication	6	I often think about how the other person perceives a situation.
6	Acceptance	6	I accept it when other cultures practice their traditions.
7	Flexibility	7	I love surprises.
8	Reflection	7	I'm okay with being unsure sometimes.
9	Tolerance of ambiguity	7	I don't like confusing situations.
10	Self-confidence	4	Other people tell me I seem confident.
11	Extraversion	3	I find it easy to make small talk.
12	Work	6	I think it's good when private and professional life are strictly separated.
13	Rule orientation	5	In Germany, pedestrians are supposed to stop at red lights, even if no cars are approaching.
14	Culturally specific knowledge	5	I don't understand table manners in Germany.
15	Conversational behavior	6	In official greetings, it's customary in Germany to shake hands.
16	Punctuality	4	I get along well with German punctuality.
17	Gender competence	5	Men are often better suited for leadership positions than women.
18	Direct/indirect communication	4	I find it difficult to understand hints from Germans.
19	Behavioral competence - SJTs	16	You‘re on your way to an important appointment. You already know you'll be late. How would you react? •I decide to skip the appointment because being late is unacceptable. •I try to notify the people involved of my delay, for example, by phone. •I'll be late. The others will surely understand.

^*^Number of items in the long version of the test.

In summary, Cult-Euro-1 represents a significant advancement in the measurement of intercultural competence. It is a true test, not a questionnaire, combining robust theory, psychometric rigor, and practical applicability. Its relevance spans human resource management, public administration, individual support (e.g., job centers), and academic research.

While the Cult-Euro-1 Test presents a novel approach to assessing both general and culture-specific intercultural competence, several limitations should be acknowledged. First, the instrument was developed and validated within the German context, which constrains the generalizability of the culture-specific dimensions to other national or institutional settings. Further adaptations and validations will be necessary to extend the framework to other cultural environments. The use of situational judgment tests provides strong ecological validity but also introduces context dependency; the interpretation of scenarios may vary depending on respondents' familiarity with German institutional and social norms. Moreover, while the concept of culture-specific competence is understood here as the ability to interact appropriately within specific environments, rather than as essentialist cultural traits, this distinction may be difficult to fully capture in standardized items. Finally, although the instrument demonstrated strong psychometric properties, further research is needed to replicate the factorial structure, test predictive validity, and assess its applicability in practical settings.

## 6 Conclusion

This paper provided a review of the definitions, models, and measurement approaches to Intercultural Competence from a psychological perspective. Beginning with foundational definitions of competence and interculturality, we examined perspectives in research, including the identity, learning theory, and stress approaches, and their relevance for predicting successful intercultural behavior. Core constructs such as competence, personality, and intelligence were introduced as a basis for understanding how intercultural competence can be meaningfully assessed.

Our review revealed a surplus, not a lack, of definitions, models, and instruments, largely due to the interdisciplinary nature of intercultural research and the varying research traditions and practices. This plurality often undermines empirical rigor. Many methods for assessment of intercultural competence fall short of scientific standards. Unregulated “intercultural training” often relies on superficial or decontextualized knowledge, especially in business contexts where cultural knowledge is frequently overemphasized. The gap between industry demand and scientifically validated tools has enabled questionable providers to market untested solutions as expertise.

We highlighted that the measurement of intercultural competence frequently lacks critical cultural specificity. Instruments often claim generalizability but are derived from culture-bound assumptions. The distinction between general (culture-free) and culture-specific competence is usually referenced but not systematically addressed.

To address these challenges, we introduced the Cult-Euro-1 Test, the first standardized instrument that measures both general and German culture-specific intercultural competence. Grounded in a strong theoretical model, empirically validated, and methodologically robust, the Cult-Euro-1 offers a meaningful advancement in the field. Its user-friendly, modular design supports use in business, administration, education, and research. By explicitly incorporating the perspective of immigrants to Germany, it ensures culturally fair and context-sensitive measurement.

Cult-Euro-1 thus serves as a foundation to validate the test for general intercultural competence in other cultural contexts while providing a methodological framework to develop culture specific scales for other cultural contexts. In the medium term, Cult-Euro-1 aims to enhance intercultural understanding, reduce discrimination, and promote equal rights and participation for migrants by enabling the targeted development of both general and culturally specific intercultural competence.
